# The Management of Lower Urinary Tract Dysfunction in Multiple Sclerosis

**DOI:** 10.1007/s11910-018-0857-z

**Published:** 2018-06-28

**Authors:** Jure Tornic, Jalesh N. Panicker

**Affiliations:** Department of Uro-Neurology, The National Hospital For Neurology and Neurosurgery and UCL Institute for Neurology, Queen Square, London, WC1N 3BG UK

**Keywords:** Review, Neurogenic lower urinary tract dysfunction, Neurogenic bladder treatment, Incontinence, Retention, Multiple sclerosis

## Abstract

**Purpose of Review:**

Multiple sclerosis (MS) is the most frequent neuroinflammatory disease of the central nervous system and is commonly associated with lower urinary tract (LUT) dysfunction. As a consequence, health-related quality of life is often impaired and the upper urinary tract might be at risk for damage. The aim of this review is to give an overview of current treatment options for LUT dysfunction in patients with MS.

**Recent Findings:**

The treatment is tailored to the type of dysfunction—storage or voiding dysfunction—beginning with conservative treatment options and ending with invasive therapies and surgery. Additionally, alternative options, e.g., different intravesical therapies or cannabinoids, have been evaluated in recent years with promising results.

**Summary:**

Current available therapies offer different possible treatments for LUT dysfunction in patients with MS. They address either voiding or storage dysfunction and therefore ameliorate LUT symptoms improve quality of life and protect the upper urinary tract.

## Introduction

Multiple sclerosis (MS) is the most common neuroinflammatory disease of the central nervous system and is a leading cause for lower urinary tract (LUT) dysfunction in neurological patients. LUT symptoms are reported on an average 8 years after the diagnosis of MS. However, in one out of ten patients with MS, LUT symptoms may be reported at the time of the initial MS manifestation [1]. Due to the progressive nature of MS, prevalence of LUT symptoms and dysfunction increases over time and reaches close to 100% by 10 years [2]. LUT dysfunction has a significant negative impact on quality of life (QoL) in patients with MS [3] and imposes a significant burden on national health care services in terms of resource allocation [4]. This emphasizes the importance of neuro-urological management in this highly complex patient population.

LUT dysfunction may present as problems of either urinary storage or voiding. Storage (overactive bladder, OAB) symptoms include urinary urgency, increased daytime frequency, nocturia (night-time frequency), and incontinence whereas voiding symptoms include urinary hesitancy, weak and interrupted stream, straining to urinate, double voiding, and sensation of incomplete bladder emptying after voiding. The pattern of symptoms and dysfunction is influenced by the distribution of MS lesions in the neuroaxis [5, 6]. Lesions in the subcortical white matter, brainstem, and spinal cord white matter that affect the neural network responsible for control of LUT functions in health result in neurogenic detrusor overactivity (NDO) [7, 8]. The severity of storage symptoms correlate with patients’ disability measured by the Expanded Disability Status Scale (EDSS) [6]. Moreover, a higher EDSS is associated with unfavorable urodynamic parameters that increase the risk for upper urinary tract damage [9••]. Detrusor external sphincter dyssynergia (DESD) leading to an increased bladder outlet resistance, and to a lesser extent detrusor underactivity (DU) due to impaired detrusor contractility and/or limited contraction duration, are responsible for voiding dysfunction, incomplete bladder emptying, and elevated post-void residuals (PVR) [1, 10]. Voiding dysfunction may be accentuated by iatrogenic factors such as treatment for storage dysfunction with antimuscarinic agents or intradetrusor botulinumtoxinA injections (BTX-A).

Needless to say, the chronic progressive nature of MS, its heterogeneity and patients’ expectations, highlight the importance of individually tailored treatment plans. The aim of this review is to provide an overview of current treatment options and recommendations for LUT dysfunction in patients with MS.

## LUT Dysfunction Management

**Fig. 1:**
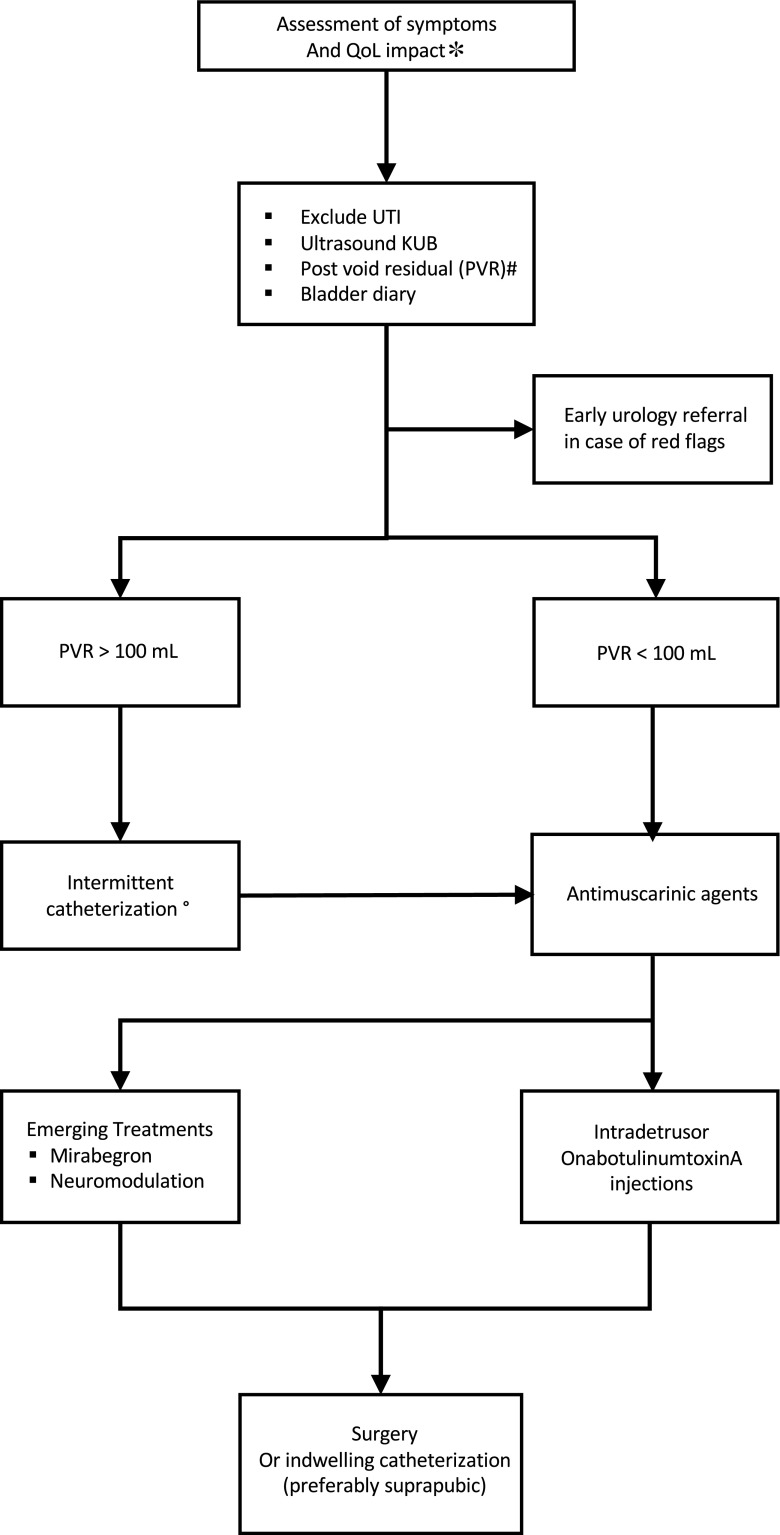
Algorithm for managing LUT dysfunction in patients with MS. Initial evaluation includes symptom and QoL assessment. UTI should be excluded and ultrasound scan KUB evaluates the upper and lower urinary tract and provides measure of the PVR. If there is a significant degree of voiding dysfunction, intermittent catheterization should be considered before treating the storage dysfunction. If initial medical treatment fails, options such as BTX-A and neuromodulation could be considered. Asterisk: additional assessment and quantification with QoL and symptom questionnaires; number sign: by ultrasound scan or in-out catheterization; degree sign: alpha1-blockers in selected cases. KUB kidney ureter bladder, PVR post void residual, QoL quality of life, UTI urinary tract infections

The management of LUT dysfunction focuses, primarily, on the improvement of patients’ symptoms and QoL and, secondarily, on the preservation of the upper urinary tract and avoidance of urological complications (e.g., urinary tract infections, bladder stones, and renal impairment). Currently, a broad armamentarium of established therapies can be offered to manage LUT dysfunction in MS. First-line treatments include fluid management, pelvic floor muscle training (PFMT), and medical therapies (e.g., antimuscarinic agents), and second-line treatments include BTX-A injections, intravesical therapies, invasive and non-invasive neuromodulation, and catheterization. Surgery may be indicated in select cases (Fig. 1). First-line management can be initiated in neurological practice, but early referral to a urology service should be considered in certain situations (Table 1).

**Table 1:** The presence of red flags should initiate an early referral to urology services

Presence of hydronephrosis	
Renal impairment	
Recurrent urinary tract infections	
Hematuria	
Suspicion of concomitant urologic pathology (e.g., prostate enlargement), stress urinary incontinence	
Loin and/or pelvic pain	
Symptoms refractory to 1st-line treatment	

## Conservative Management

### Pelvic Floor Muscle Training

Pelvic floor muscle training may be used solely, or in combination with other therapies, to treat LUT dysfunction in patients with MS. PFMT is effective in patients who demonstrate the ability to contract pelvic floor muscles. The proposed mechanism of action is through the activation of an inhibitory reflex on detrusor activity when the pelvic floor muscles are voluntarily contracted [11]. The benefits of PFMT are modest [12, 13] and studies are limited by low patient numbers and heterogeneous outcome measures. A recent study demonstrated that PFMT alone or in combination with neuromuscular electrical stimulation (NMES) or transcutaneous tibial nerve stimulation (TTNS) improved storage symptoms in women with MS [14••]. Due to its non-invasiveness, PFMT should be continued whenever possible throughout the treatment course.

### Medical Treatments

The recommendations for pharmacological treatment of LUT dysfunction in neurological patients depend on whether the patient has storage (overactive bladder) or voiding (underactive bladder or detrusor sphincter dyssynergia) problems but are specifically selected often based on anecdotal experience, expert opinion, and extrapolation of the results from clinical trials in non-neurological patients [4, 15–17]. For storage problems, antimuscarinics are the first-line option, and more recently, beta-3-receptor agonists have become available and can be useful either as an add-on or stand-alone treatment. Antimuscarinics with a beneficial neurologic side effect profile such as darifenacin or trospium (see the “Antimuscarinics” section) can be a good choice in patients with neurological disease such as MS. To observe how patients might tolerate a medication, treatment should be initiated with the lowest dose, which can subsequently be increased depending on patients’ symptoms and occurrence of medication-related side effects. There is no clear consensus on how long antimuscarinics should be given before assessing their efficacy. However, there is one study assessing efficacy of solifenacin and tolterodine in patients with idiopathic OAB where the reported median time for a therapeutic response was 3 months [18]. Usually, two different antimuscarinics are trialed before the patient is considered to be refractory to medical therapy and second-line treatments—including BTX-A intradetrusor injections and neuromodulation—may be evaluated (Fig. 1).

For voiding problems, only alpha-blockers are currently considered for medical therapy, and in refractory cases neuromodulation or catheterization, preferably clean intermittent catheterization (see the “Catheterization” section) can be offered. However, there are no recommendations for the treatment of the underlying causes DESD and DU.

#### Antimuscarinics

Different antimuscarinic medications are available for managing LUT symptoms, with similar efficacy and treatment outcomes (Table 2). They reduce NDO and OAB symptoms by blocking muscarinic receptors distributed throughout the detrusor and suburothelium, thus blocking parasympathetic-mediated activation of the detrusor [19]. The M3 muscarinic receptor is of greatest significance functionally; however, most of the antimuscarinics non-selectively bind with muscarinic receptors of different sub-types across several organs. This is responsible for the side effect profile of these medications including dry mouth, blurred vision, and constipation [20], which influence adherence to these medications [20, 21].

**Table 2:** Currently available antimuscarinic agents for the treatment of neurogenic LUT dysfunction

Agent	Release type	Dose (mg)	Frequency	Level of evidence for treatment of neurogenic LUT dysfunction
Darifenacin	Controlled release	7.5–15	Once daily	NA
Fesoterodine	Controlled release	4–8	Once daily	NA
Oxybutynin	Immediate release	2.5–5	2–3 times daily	1 [22]
	Controlled release	5–20	Once daily	1 [22]
	Transdermal patch	36	Replace once every 3–4 days	1 [22]
Propiverine	Immediate release	15	1–3 times daily	1 [23]
	Controlled release	30	Once daily	1 [23]
Solifenacin	Controlled release	5–10	Once daily	2 [24]
Tolterodine	Immediate release	2–4	1–2 times daily	3 [25]
	Controlled release	4	Once daily	3 [25]
Trospium chloride	Immediate release	20	Twice daily	1 [26]
	Controlled release	60	Once daily	1 [26]

*NA* not available

A recently published double-blind, randomized controlled trial (RCT) comparing two different antimuscarinics, oxybutynin and solifenacin, to placebo demonstrated significant improvements in urinary frequency and incontinence, as well as QoL with both medications. Additionally, urodynamic parameters such as the maximum cystometric capacity also improved. The most common reported side effect was dry mouth, more often affecting patients using oxybutynin than solifenacin [27••]. To avoid side effects and/or simultaneously improve treatment efficacy, the combination of an antimuscarinic with mirabegron or an alpha1-blocker (e.g., tamsulosin), particularly in men with additional prostate-related voiding dysfunction, might be explored. However, the current evidence of such combinations is exclusively based on data from patients with idiopathic LUT dysfunction [28, 29].

Meta-analyses of studies evaluating the efficacy of antimuscarinics in neurological patients have shown no significant differences in efficacy between individual agents [20]. Rather, the extent of side effects is considerably less among newer antimuscarinics such as solifenacin, tolterodine, and fesoterodine and therefore these are preferred in general. The use of medications with anticholinergic properties has been linked with incidental dementia in an epidemiological study [30], linking their use with poorer cognition, reduced cerebral glucose metabolism, increased brain atrophy, and greater clinical decline [31••]. This is of particular relevance in MS where cognitive impairment can affect 43–65% of patients [32, 33]. In patients where cognitive impairment is a concern, trospium chloride is an option to consider because its quaternary amine structure renders it relatively impermeable to the blood-brain barrier [34, 35]. Alternatively, darifenacin may be considered in view of its high selectivity for the M3 muscarinic receptors in the bladder [36].

However, despite these concerns, antimuscarinic agents continue to be the first-line treatment for OAB in neurological patients due to their favorable cost-benefit ratio [4].

#### Mirabegron

Mirabegron is a beta-3-receptor agonist and therefore works differently from antimuscarinics. In non-neurologic patients with bladder storage dysfunction, mirabegron is associated with significant improvements in incontinence episodes and urinary frequency [37]. However, data on efficacy and safety in MS are limited. Zachariou et al. demonstrated in a recently published open-label study that mirabegron and desmopressin, either alone or in combination, significantly improved urinary urgency, frequency, and incontinence episodes [38••].

Possible side effects include hypertension, tachycardia, and headache [37]. Despite the limited evidence base in neurogenic OAB, mirabegron is increasingly being used as an alternative to or in combination with antimuscarinics.

#### Alpha1-blockers

Alpha1-adrenergic blockers exert an inhibitory effect on the sympathetic innervation of the smooth muscle of the bladder neck and internal urethral sphincter, thereby reducing the extent of bladder outlet obstruction [39•]. Data on the efficacy and safety of alpha1-blockers to treat neurogenic bladder dysfunction are limited [40, 41, 42•], and the only published study, specifically in MS, demonstrated a significant improvement in voiding parameters including peak flow rate [43]. Alpha1-blockers are a well-established first-line treatment for male patients with voiding dysfunction due to benign prostate enlargement and can be considered as an option in men with MS reporting significant voiding dysfunction [4, 44, 45]. Moreover, they can be combined with antimuscarinics in patients with concomitant storage symptoms (see the “Antimuscarinics” section). Most common side effects are retrograde ejaculation and orthostatic dysregulation, which are reversible.

#### Desmopressin

Desmopressin is a synthetic vasopressin (antidiuretic hormone, ADH) analogue which enhances water reabsorption in the renal collecting duct by upregulation of water channels (aquaporin II) via V2-receptors [46]. As a result, urine volume decreases so that patients with diabetes insipidus, nocturnal enuresis, and nocturia can be treated.

The results from a meta-analysis of studies in MS published in 2005 demonstrated that desmopressin reduced daytime frequency, urine volume, and sleep efficiency by prolonging the duration of uninterrupted sleep by an average of 2 h. Desmopressin is effective for the treatment of nocturia due to MS-related NDO with maximum bladder capacity being a clinically useful predictor of treatment response [47]. Desmopressin has been shown to improve urinary frequency, urgency, incontinence episodes, and pad usage, and the effects were accentuated when combined with mirabegron [38••].

The most common side effect is hyponatremia (8%) [48], followed by urinary retention (0–8%) and headache (3–4%) [49]. Patients at increased risk for hyponatremia are females, elderly patients, and patients with concomitant cardiac disease or elevated 24-h urine volume [50, 51]. In those patient groups, sodium levels should be monitored periodically when using desmopressin, especially in the initial phase [52]. A suggested monitoring plan by Juul et al. includes baseline sodium monitoring, which should be ≥ 135 mmol/L, and additional monitoring at week 1 and month 1 particularly in patients at high risk for developing hyponatremia [53]. Moreover, it is also well known that long-term administration of desmopressin might slowly lower sodium serum levels over time. Therefore, serum sodium should be periodically monitored every 6 months [54]. To reduce the risk for developing hyponatremia, desmopressin should be administered only once in 24 h, and fluid intake should be limited during the 6–8-h period of effect.

#### Phosphodiesterase Type 5 Inhibitors

Phosphodiesterase type 5 inhibitors (PDE5Is) regulate smooth muscle tone via the nitric oxide (NO) pathway and are recommended as first-line on demand treatment for neurogenic erectile dysfunction (ED) in patients with MS [55, 56]. However, they have also been shown to be effective for managing OAB [57••]. Tadalafil 5 mg daily significantly improved subjective storage and voiding symptoms and additionally had a positive impact on maximum flow rate and PVR in a small cohort of 20 young male MS patients, besides having a positive effect on erectile functions [57••]; however, no studies are available in women. In general, PDE5Is are a promising option for managing MS-related urogenital dysfunction, although recommendations for its use currently do not exist.

#### Cannabinoids

Cannabinoid receptors play a significant role in sensory nerve signaling, bladder afferent functions, and possibly modulation of cholinergic nerves [58]. Cannabinoid preparations reduce detrusor contractility possibly through cannabinoid receptors [59, 60] distributed in the detrusor and the central nervous system [61]. Recreational use of inhaled cannabis has been shown to improve LUT symptoms in MS [62]. In a recent systematic review, efficacy and safety of cannabinoids for the treatment of LUT dysfunction in patients with MS was evaluated. Despite the low quality of existing evidence due to heterogeneity in reported outcomes, small numbers of patients studied, and insufficient follow-up, the evidence suggests that treating LUT dysfunction might be effective with a favorable safety profile [63••]. However, cannabinoids are currently not licensed for treating LUT dysfunction, are illegal in the USA according to federal law, and not reimbursed in several countries.

## Intravesical Treatments

### BotulinumtoxinA

OnabotulinumtoxinA (Botox, Allergan) and abobotulinumtoxinA (Dysport, Ipsen Biopharm Ltd.) are used for the treatment of NDO. A comparative study between these molecules is lacking, though outcomes appear to be similar when used in animal models [64].

Intradetrusor onabotulinumtoxinA has become a well-established second-line treatment for NDO. Injections are administered via cystoscopy under local or general anesthesia. Two pivotal phase 3 studies reported that 200 and 300 IU onabotulinumtoxinA significantly reduces urinary incontinence episodes and improves QoL and urodynamic parameters in patients with MS and spinal cord injury (SCI) [65, 66]. Clinically significant benefits were observed after 6 weeks and 60% of patients reported a significant reduction in weekly urinary incontinence episodes and improvement of QoL using both dosages compared to placebo. Botulinum toxin inhibits detrusor activity and thereby is associated with developing urinary retention. De novo initiation of clean intermittent catheterization (CIC) was significantly different between the two groups, i.e., 30 versus 42% in the 200 and 300 IU groups, respectively, compared to 12% in the placebo group [65]. However, there were no significant differences in efficacy or duration of effect between the two dosages. Moreover, both dosages were well tolerated with a more favorable safety profile, i.e., UTI rate, in the 200 IU group. For these reasons, 200 IU onabotulinumtoxinA was approved by the US Food and Drug Administration (FDA) in 2011 for treating NDO and is licensed in Europe for the treatment of NDO in MS and SCI. Most frequent reported adverse events after onabotulinumtoxinA intradetrusor injections include urinary retention and UTIs in 52 and 56%, respectively [67]. OnabotulinumtoxinA has proven efficacy over long-term repeat injections [68••] with consistent inter-injection intervals [69]. However, discontinuation of treatment is a relevant issue and Leitner et al. showed in a consecutive series of patients with LUT dysfunction due to different neurological conditions with a follow-up of up to 17 years that approximately 40% of patients discontinue treatment over time. Reasons for discontinuation were lack of clinical and/or urodynamic effects, or preference of another treatment such as neuromodulation or bladder augmentation despite objective evidence for efficacy. More than half of patients in the MS subgroup discontinued treatment due to progression of MS and progressive loss of responsiveness to botulinumtoxinA (BTX-A) [70••].

In a retrospective study with a mixed group of neurological patients with refractory LUT dysfunction using intradetrusor injections of 750 IU abobotulinumtoxinA, 64% of patients reported significantly improved continence rates after 6 weeks and a reduction in mean maximum detrusor pressures comparable to after onabotulinumtoxinA use. The study confirmed long-time safety and efficacy of abobotulinumtoxinA 750 IU over a mean follow-up of 28 months [71••].

### Other Intravesical Treatments

Intravesical administration of active pharmaceutical ingredients might be considered to avoid systemic side effects because of different metabolic pathways [72]. In a recently published systematic review on intravesical administration of vanilloids (capsaicin and resiniferatoxin) in MS, intravesical vanilloid instillation was shown to be effective for treating LUT dysfunction. However, the safety profile was unfavorable with reports of pelvic pain, urinary tract infection, and hematuria reported by > 50% of the patients [73]. Therefore, no current high-quality evidence has been published to support the use of vanilloids for intravesical therapy in patients with MS. Moreover, clinical studies have shown that resiniferatoxin has limited clinical efficacy compared to BTX-A injections in the detrusor [74].

Studies have evaluated intravesical administration of antimuscarinic agents and shown that the use of intravesical oxybutynin is efficacious and safe [75•, 76, 77]. There is no general consensus on cumulative dose and administration frequency. Schroder et al. used a protocol with intravesical administration of 10 mL oxybutynin hydrochloride 0.1% (10 mg oxybutynin hydrochloride/10 mL 0.9% saline) three times daily (cumulative dose 30 mg/day) directly into an emptied bladder through a urethral catheter [75•].

Since all currently existing studies evaluate intravesical oxybutynin only in patients with SCI or spina bifida, recommendations for its use in MS cannot be made. However, since any intravesical administration requires catheterization, acceptance of this form of therapy might be greater in patients already performing CIC than in patients spontaneously voiding [77].

## Neuromodulation

### Tibial Nerve Stimulation

Percutaneous and transcutaneous tibial nerve stimulation (PTNS and TTNS, respectively) have been shown to be beneficial in the management of OAB. Stimulation is performed by introducing a needle electrode (PTNS) or cutaneous patch electrode (TTNS) over the course of the tibial nerve, approximately 5 cm cephalad and posterior to the medial malleolus. The treatment schedule conventionally adopted is intermittent 30 -min stimulation sessions over 12 sessions. There is no evidence favoring one schedule over the other [78]. Schneider et al. assessed efficacy and safety of both treatments in a systematic review with meta-analysis [79••]. The results were promising and showed that PTNS and TTNS might be effective and safe for the treatment of neurogenic LUT dysfunction. However, the quality of included studies was low, with only a few studies evaluating patients with MS.

TTNS has been shown to be an effective treatment. In a prospective open-label study, de Sèze et al. demonstrated through a pre-post treatment design that urgency, leakages, QoL, and urodynamic parameters significantly improved after daily TTNS treatment for 3 months [80]. Additionally, Kabay et al. reported that a durable effect could be achieved with a tapering protocol over 12 months in treatment responders [81••]. The effects of tibial nerve stimulation are comparable to antimuscarinic agents in the non-neurological population [82]; however, how this treatment compares with BTX-A has never been evaluated.

### Sacral Neuromodulation

In contrast to tibial nerve stimulation, sacral neuromodulation (SNM) is a surgical treatment implanting a stimulation electrode in the sacral foramen S3 connected to a battery device, which is usually placed in the upper buttock [83].

In a systematic review by Kessler et al. assessing efficacy of SNM in neurogenic patients, subgroup analysis of patients with MS showed a success rate of 84%, defined as an improvement of > 50% in different variables such as number of leakages, pad use, number of voids, and number of catheterizations using a bladder diary [84, 85]. Overall evidence indicates that SNM may be effective and safe in patients with LUT dysfunction. However, the conclusion is limited due to small number of patients studied and low quality and heterogeneous study designs [86]. The success rate is consistent with an earlier published retrospective cohort study, which reported significant reductions in day and night-time frequency, incontinence episodes, pad usage, and number of CICs per 24 h in a mixed neurological patient group with almost 50% of patients having MS [87].

However, in view of the progressive course of neurological and urological disability, the benefits of SNM may be lost over time [70••]. Moreover, according to the manufacturer, the currently available device InterStim II (Medtronic, Inc., Minneapolis, MN, USA) is MR incompatible, and therefore, using this device would not be an option in patients requiring repeat magnetic resonance (MR) imaging. Elkelini and Hassouna, however, have reported a series of eight patients with implanted SNM undergoing MR imaging at 1.5 Tesla without safety concerns [88].

In conclusion, there is evidence suggesting effectivity of SNM for treating LUT dysfunction but due to the lack of well-designed RCTs, no final recommendations can be made. A double-blind RCT in patients with LUT dysfunction due to different neurological conditions (ClinicalTrials.gov NCT02165774) is however underway [89].

## Surgical Options

In select MS patients with LUT dysfunction refractory to first- and second-line treatments, surgery might need to be considered as an option. The risk for developing upper urinary tract damage is low compared to other neurological conditions such as SCI, and the consideration for surgery has further declined over the years in view of the availability of effective first- and second-line treatments. The role for surgery in the management of MS-related LUT dysfunction is therefore limited [90]. Surgery might be a valuable option particularly in young individuals with anticipated long-life expectancy and patients with substantial impairment in QoL due to refractory LUT dysfunction. Surgical options that might be offered include bladder augmentation, cystectomy, and continent and incontinent urinary diversion. The choice of surgery is influenced to a large extent by motor functions and cognitive abilities, and patient expectations. However, as MS is a progressive disease with likely deterioration of these neurological disabilities and inability to catheterize over time, an incontinent urinary diversion such as an ileal conduit is often preferred [91].

A prospective study evaluating MS patients with advanced disease showed that laparoscopic-assisted cystectomy with ileal conduit was associated with significant improvement in QoL domains such as limitations, constraints, and specific urinary impact index using the Qualiveen questionnaire, though the overall score remained unchanged. A higher complication rate was reported in patients with longer disease duration, suggesting that surgical management should be considered as a possibility early in patients refractory to first- and second-line treatments who are likely to progress [92].

Women with pelvic floor insufficiency reporting stress urinary incontinence (SUI) may be offered a mid-urethral sling in select cases; however, they should undergo cystoscopy and urodynamic study to assess the risk of developing urinary retention post-operatively [90].

## Catheterization

Depending on the pattern and extent of LUT dysfunction and patients’ disability level, intermittent or indwelling catheterization may be offered to address the problem of incomplete bladder emptying. Most MS patients present with storage dysfunction [2]; however, voiding dysfunction and urinary retention occur in up to 70% [93]. Moreover, managing storage dysfunction with antimuscarinics or intradetrusor BTX-A injections is often associated with worsening voiding.

Since the introduction of clean intermittent catheterization (CIC) in a patient with MS by Lapides et al. in 1972, CIC has become the gold standard in the treatment of voiding dysfunction [94]. One prospective cohort study demonstrated a significant impact in bladder-specific QoL using the Qualiveen questionnaire in patients with MS reporting LUT dysfunction [95]. A substantial improvement in QoL and LUT symptoms of urinary frequency, urgency, stress, and urgency incontinence were reported in a cross-sectional study [96]. Additionally, regular CIC appears to improve PVR over time in MS patients who are voiding spontaneously, suggesting that regular complete bladder emptying avoiding overdistension might improve bladder functions [97].

The risk of developing UTIs is a concern; however, compared to other bladder-emptying methods including the use of indwelling transurethral or suprapubic catheters, Crédé maneuver, and reflex micturition, CIC has been reported to decrease long-term urinary tract complications and improve QoL in patients with neurogenic LUT dysfunction [98, 99]. In regard to patients with MS, Luoto et al. reported a higher incidence of UTIs after initiation of CIC; however, this did not reach statistical significance. Moreover, infections were associated with less subjective discomfort compared to before commencing catheterization [96]. Additionally, Andretta et al. reported serious complications in 2 out of 10 patients with MS (20%): one male patient developed epididymorchitis and another patient was found to have a bladder stone [100].

As mentioned above, compared to intermittent catheterization, indwelling catheters are associated with a greater risk for complications such as UTIs, genital erosions, and stone formation. Should long-term indwelling catheterization be considered, the suprapubic route is preferable in view of patient comfort, facilitation of intimacy, easier handling, and lesser complications. In the context of long-term indwelling catheterization, silicone rather than latex catheters should be used as they are associated with less susceptibility for encrustation and allergy in the neurological population [101].

In conclusion, CIC is preferred over indwelling catheterization (grade A recommendation) as a standard treatment for patients with LUT dysfunction who are unable to empty their bladder despite the low level of evidence (LOE 3) [4]. There are no specific recommendations for patients with MS.

## Conclusions

Neurogenic lower urinary tract dysfunction is common in patients with MS and is associated with a significant negative impact on quality of life. The management of bladder dysfunction is individually tailored according to the pattern of LUT dysfunction, extent of neurological disabilities, disease course, and patient expectations. Treatment remains a challenge for health care providers; however, adopting a treatment algorithm beginning with non-invasive therapy offers a wide spectrum of different treatments targeting different mechanisms for managing LUT dysfunction.
